# Ferroptosis-Related Gene Signature and Patterns of Immune Infiltration Predict the Overall Survival in Patients With Lung Adenocarcinoma

**DOI:** 10.3389/fmolb.2021.692530

**Published:** 2021-07-30

**Authors:** Yuxuan Wang, Weikang Chen, Minqi Zhu, Lei Xian

**Affiliations:** ^1^ Guangxi Medical University, Nanning, China; ^2^ Department of Cardiothoracic Surgery, The Second Affiliated Hospital of Guangxi Medical University, Thoracic and Cardiovascular Surgery, Nanning, China

**Keywords:** lung adenocarcinoma, ferroptosis, gene signature, overall survival, immune status

## Abstract

**Background:** Lung adenocarcinoma (LUAD) is a malignant tumor with high heterogeneity and poor prognosis. Ferroptosis, a form of regulated cell-death–related iron, has been proven to trigger inflammation-associated immunosuppression in the tumor microenvironment, which promotes tumor growth. Therefore, the clinical prognostic value of ferroptosis-related genes in LUAD needs to be further explored.

**Method:** In this study, we downloaded the mRNA expression profiles and corresponding clinical data of LUAD patients from the Cancer Genome Atlas database. The least absolute shrinkage and selection operator (LASSO) Cox regression model was utilized to construct ferroptosis-related gene signature. Based on these, we established the nomograms for prognosis prediction and validated the model in the GSE72094 dataset. The cell type was identified using the CIBERSORT algorithm for estimating relative subsets of RNA transcripts, which was then used to screen significant tumor immune-infiltrating cells associated with the LUAD prognosis prediction model. Subsequently, we applied co-expression analysis to reveal the relationship between ferroptosis-related genes and significant immune cells.

**Results:** The univariate COX regression analysis showed that 20 genes were associated with the overall survival (OS) as prognostic differentially expressed genes (DEGs) (FDR <0.05). Patients were divided into two risk groups using a 13-gene signature, with the high-risk group having a significantly worse OS than their low-risk counterparts (*p* < 0.001). We used receiver operating characteristic (ROC) curve analysis to confirm the predictive capacity of the signature. Besides, we identified seven pairs of ferroptosis-related genes and tumor-infiltrating immune cells associated with the prognosis of LUAD patients.

**Conclusion:** In this study, we construct a ferroptosis-related gene signature that can be used for prognostic prediction in LUAD. In addition, we reveal a potential connection between ferroptosis and tumor-infiltrating immune cells.

## Introduction

Lung cancer is the most common malignant tumor in the world and has become the leading cause of death among other cancers. According to the data, there would be 2.2 million new cases of lung cancer and 1.8 million deaths worldwide in 2020, making lung cancer rank first in terms of incidence and mortality of malignant tumors (Siegel et al., 2021). Non–small-cell lung cancer (NSCLC) is the most common type of lung cancer, accounting for more than 80% of all lung cancer patients (Ettinger et al., 2017). Examples of NSCLC include adenocarcinoma, squamous cell carcinoma, and large-cell carcinoma. Lung adenocarcinoma (LUAD) is a major histologic type of NSCLC, accounting for approximately 50% of all cases (Travis et al., 2015). Despite advances in surgery, chemotherapy, radiotherapy, targeted therapy, immunotherapy, and other forms of treatment, the overall five-year survival rate for NSCLC patients remains less than 25% (Ettinger et al., 2017). Thus, considering the poor prognosis of LUAD patients, it is imperative to develop a novel prognostic model. Prognostic prediction models involve the prediction of the probability or risk of a particular outcome or event in the future, such as prediction of recurrence or death after diagnosis of cancer or mortality after surgery (Moons et al., 2014).

Regulated cell death (RCD) is one of the main distinct categories of cell death, which include apoptosis and autophagy. RCD, which relies on dedicated molecular machinery, has an obvious advantage for organismal homeostasis in setting. Ferroptosis is a form of non-apoptotic regulated cell death, which is characterized by overproduction of lipid peroxidation and lethal accumulation of reactive oxygen species (ROS) (Stockwell et al., 2017). Ferroptosis has recently gained popularity as a therapeutic alternative for malignancies that are resistant to traditional treatments (Chen et al., 2021). As more ferroptosis-related studies emerge, numerous genes have been identified as modulators or markers of ferroptosis. Research on cancer has discovered that ferroptosis is involved in multiple tumor biological processes, such as immune escape, invasion, and metastasis (Jiang et al., 2021). Particularly, recent research proved that CD36-mediated ferroptosis can reduce the effects of CD8 T cell in promoting tumorigenesis (Ma et al., 2021). Other studies have linked ferroptosis to lung cancer, but it is unclear whether some genes that regulate ferroptosis, such as P53 (Zhu et al., 2021), GPX4 (Zhang et al., 2021), and SLC7A11 (Hong et al., 2021), are correlated with the prognosis of LUAD patients.

Lung adenocarcinoma, as a solid tumor, forms a complex ecosystem including normal epithelial cells, fibroblasts, and infiltrating immune cells, among others, in addition to accumulating cancer cells (Remark et al., 2015; Ahmed, 2019). Tumor-infiltrating immune cells are part of tumor microenvironment (TME), which regulates tumor growth and development. Increasing evidence has proved that these immune cells play a crucial role in the development and progression of cancers (Chen et al., 2019), and in tumor response to therapy (Dunn et al., 2004; Fridman et al., 2012). According to certain researchers, infiltrating immune cells should be considered when determining the prognosis of cancer patients (Broussard and Disis, 2011). Evidence has shown that B-cell infiltration in lung cancer is significantly higher than that in nontumoral tissue (Del mar valenzuela-membrives et al., 2016). Therefore, developing a prognostic prediction model based on infiltrating immune cells will be valuable to clinicians.

In the present study, the mRNA expression profiles and relevant clinical data of patients with LUAD were downloaded from the TCGA database. Subsequently, the prognostic multigene signature of ferroptosis-related differentially expressed genes (DEGs) was constructed. The Cibersort (estimating relative subsets of RNA transcripts) algorithm was applied to establish a nomogram of the type of tumor-infiltrating immune cells. Finally, we utilized co-expression analysis to reveal the underlying mechanisms between ferroptosis-related genes and tumor-infiltrating immune cells.

## Materials and Methods

### Data Collection

Data that contained RNA expression profiles and relevant clinical information of 535 lung adenocarcinoma patients were downloaded from the Cancer Genome Atlas (TCGA) database (https://portal.gdc.cancer.gov/). The gene expression profiles were normalized using the scale method in the R package “limma.” The raw data of mRNA expression matrix of GSE72094 were downloaded from the GEO database (Gene Expression Omnibus, https://www.ncbi.nlm.nih.gov/geo/query/acc.cgi). The platform of GSE72094 was GPL15048 [Rosetta/Merck Human RSTA Custom Affymetrix 2.0 microarray (HuRSTA_2a520709. CDF)]. Count data were normalized. Besides, corresponding clinical information of all patients was also collected, such as age, gender, TNM stage, survival time, and survival status. Cases with no information on survival status and survival time would be deleted. Since the TCGA data and GEO data are publicly available, our research did not require the approval of local ethics committees. However, the present study adhered to the data access policies and publication guidelines provided by TCGA and GEO.

The ferroptosis-related genes were collected from the previous literature (Liang et al., 2020) and the FerrDb database (Zhou and Bao, 2020) (Supplementary Table 1).

### Construction and Validation of the Prognostic Signature of Ferroptosis-Related Genes

The “limma” R package was used to analyze the differentially expressed genes (DEGs) between tumor tissues and adjacent nontumorous tissues with a false discovery rate (FDR) < 0.05. Univariate Cox analysis of the overall survival (OS) was used to screen prognostic values of ferroptosis-related genes. *p*-values were adjusted using the Benjamini and Hochberg (BH) method. An interaction network of the overlapping genes of prognostic genes and DEGs was constructed using the STRING database (version 11.0) (SzklarczyK et al., 2011). The prognostic model generated by the LASSO-penalized Cox regression analysis was used to minimize the risk of overfitting (TIbshirani, 1997; Simon et al., 2011), whereas the “glmnet” R package was used to select and shrink variables of the LASSO algorithm. The normalized expression matrix of candidate prognostic DEGs was the independent variable in the regression, whereas the overall survival and status of patients were the response variables. In this model, we used tenfold cross-validation to determine the penalty parameter (λ) based on the minimum criterion (i.e., the value of λ corresponding to the lowest partial likelihood deviance). The normalized expression levels of each gene and its corresponding regression coefficients were used to calculate the risk scores of the patients. The formula (Liang et al., 2020) was established as follows: score = e^sum (each gene’s expression × corresponding coefficient)^. We divided patients into high-risk and low-risk groups according to the median risk score. The “stats” R package was used to perform PCA based on the expression of genes in the signature. Finally, we determined the optimal cutoff expression value of the survival analysis by the “surv_cutpoint” function of the “survminer” R package; whereas the time‐dependent ROC curve analysis was carried out using the “survivalROC” R package. The validation cohort was constructed based on the GSE72049 dataset, and the constructed nomogram model was validated by the validation cohort using the same process of the TCGA cohort with the ROC analysis and Kaplan–Meier curve.

### Functional Enrichment Analysis

We used the “clusterProfiler” R package to perform Gene Ontology (GO) and Kyoto Encyclopedia of Genes and Genomes (KEGG) analyses between the high-risk and low-risk groups, based on the DEGs (|log2FC| ≥ 1, FDR <0.05). *p*-values were adjusted with the BH method. The single-sample gene set enrichment analysis (ssGSEA) of the infiltrating score of 16 immune cells and the activity of 13 immune-related pathways were calculated in the “gsva” R package (Rooney et al., 2015).

### CIBERSORT Estimation

We used CIBERSORT, an analytical algorithm that deconvolutes bulk tumor samples with a minimal representation for each cell type using support vector regression based on a set of reference gene expression values. CIBERSORT analyzes RNA expression data to evaluate the abundance of different cell subtypes in each sample, to examine 22 immune cell types of lung adenocarcinoma and to estimate the proportion of infiltrating immune cells. Only samples with a CIBERSORT output of *p* < 0.05 were considered worthy of further analysis. The Wilcoxon rank sum test showed a significant difference in the proportion of the immune-infiltrating cells in tumor tissues and adjacent nontumorous tissues.

### Construction of the Prognostic Tumor Immune Cells

We used Kaplan–Meier survival analysis and Cox regression to detect the prognosis-associated cell types. To ensure that the multifactor models were not overfitting, we integrated all significant cells into the Cox model and then constructed a simplified Cox model. The ROC and area under the curve (AUC) were quantified to assess the sensitivity and specificity of our diagnostic and prognostic models. The accuracy of the predictive capacity of the nomogram was evaluated using a calibration curve and a consistency index. Finally, the relationship between ferroptosis-related gene signature and the 22 types of immune cells was investigated using the Pearson correlation coefficient (visualized by co-expression heat map). In addition, decision curve analysis was performed to determine the clinical utility.

### Statistical Analysis

Statistical significance was determined by a two-sided *p* <0.05, except when a special explanation was given. All statistical analyses were carried out in R software (version 3.6.3) using the following software packages: limma, ggplot2, rms, glmnet, survminer, clusterProfiler, gsva, and timeROC.

## Results

### Identification of Prognostic Ferroptosis-Related DEGs

**FIGURE 1:**
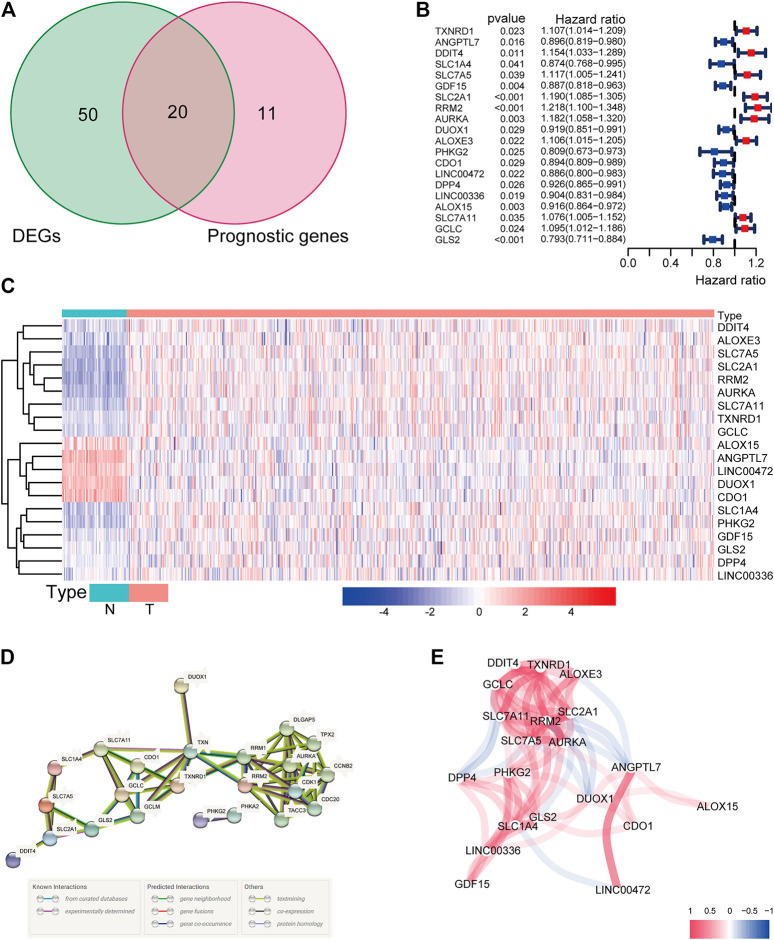
**(A)** Venn diagram to identify differentially expressed genes between tumor and adjacent normal tissue that were correlated with OS. **(B)** Forest plots showing the results of the univariate Cox regression analysis between gene expression and OS. **(C)** The expression of these 20 overlapping genes in tumor tissue and normal tissue. **(D)** The PPI network downloaded from the STRING database indicated the interactions among the candidate genes. **(E)** The correlation network of candidate genes. The correlation coefficients are represented by different colors.

There were 71 ferroptosis-related genes that were differentially expressed between tumor tissues and adjacent nontumorous tissues. We preserved 20 genes correlated with OS as prognostic DEGs (all FDR < 0.05) based on the univariate COX regression analysis (Figures 1A–C). The interaction network among these genes indicated that RRM2, AURKA, TXNRD1, and RRM1 were the hub genes. The correlations between these genes were shown in Figures 1D,E.

### Construction and Validation of a Prognostic Model

**FIGURE 2:**
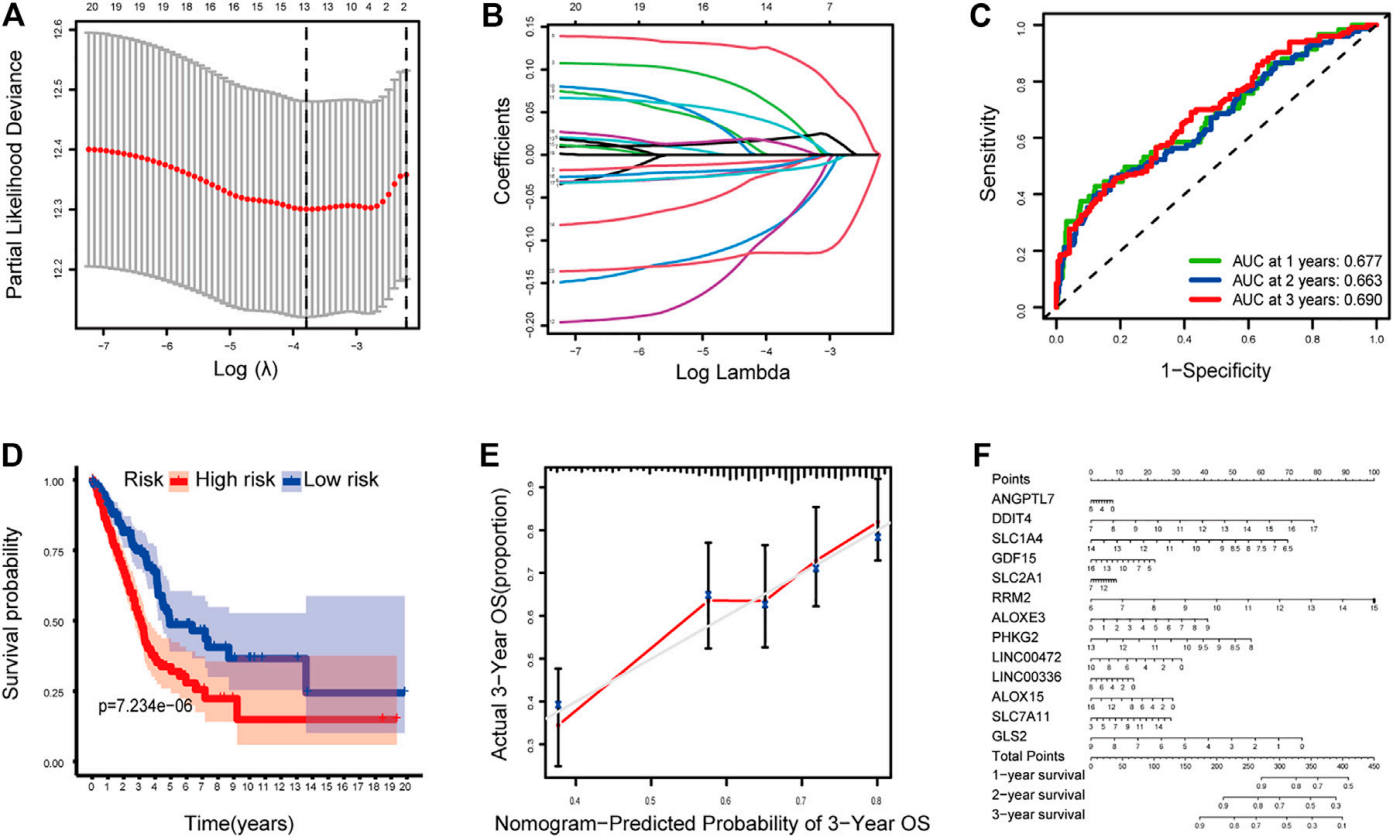
**(A–B)** Model diagnosis process Lasso regression curves which were constructed by the Ferroptosis-related genes signature. **(C)** AUC of timedependent ROC curves verified the predicting prognostic performance. **(D)** Kaplan–Meier curves for the OS of patients in the high-risk group and low-risk group. **(E-F)** The calibration curves and nomogram. ROC, receiver operating characteristic curve. *p < 0.05.

The LASSO Cox regression analysis was applied to construct a prognostic model based on the expression profiles of the 20 previously analyzed genes. A 13-gene signature was identified based on the optimal value of lambda. The 13 genes which included ANGPTL7, DDIT4, SLC1A4, GDF15, SLC2A1, RRM2, ALOXE3, PHKG2, LINC00472, LINC00336, ALOX15, SLC7A11, and GLS2 were used to construct a multiple Cox risk regression model (Figures 2A,B). In addition, a nomogram was constructed using the 13 selected genes, and utilized to predict a 1-, 2-, and 3-year survival status. The calibration curves showed that the nomogram performed well (Figures 2E,F, Supplementary Figures 3, 4).

**FIGURE 3:**
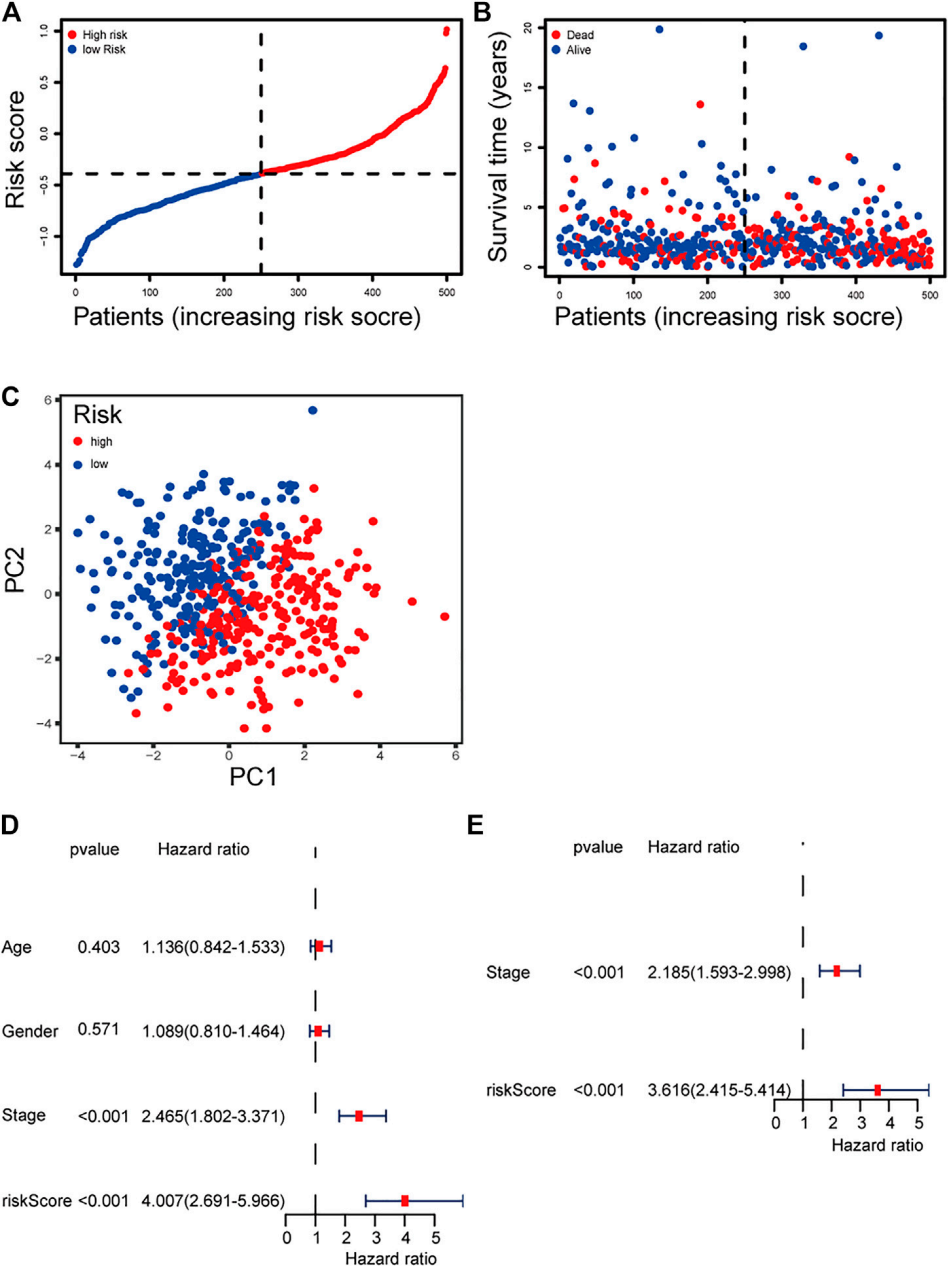
**(A)** Distribution and median value of the risk scores of the ferroptosis-related genes signature. **(B)** The distributions of OS status, OS, and risk score of the ferroptosis-related genes signature. **(C)** PCA plot of the ferroptosis-related genes signature. **(D–E)** Results of the univariate and multivariate Cox regression analyses regarding OS of the ferroptosis-related genes signature.

The patients were divided into a high-risk group (*n* = 250) and a low-risk group (*n* = 250) according to the median cutoff value. The patients in the different risk groups were distributed in two directions based on the PCA analysis (Figures 3A–C). The Kaplan–Meier curve showed that patients with a high risk had a significantly worse OS, and thus were more likely to die earlier than those with the low risk (*p* < 0.001; Figure 2D). The time-dependent ROC curves and the area under the curve (AUC) indicated a score of 0.677 at 1 year, 0.663 at 2 years, 0.690 at 3 years (Figure 2C). The GSE72094 dataset was used as a validation cohort to validate the prognostic model. The Kaplan–Meier curve, the time-dependent ROC curves, and the area under the curve (AUC) based on validation cohort are performed in Supplementary Figure 2.

### Independent Prognostic Value of the 13-Gene Signature

We performed univariate and multivariate Cox regression analyses on clinical variables to determine whether the risk score was an independent prognostic predictor of OS. We realized that the risk score was significantly associated with OS in univariate Cox regression analysis (HR = 4.007; 95% CI = 2.691–5.966; *p* < 0.001; Figure 3E), and that despite correcting other confounding factors in the multivariate Cox regression analysis, the risk score was nevertheless proved to be an independent predictor for OS (HR = 3.616; 95% CI = 2.415–5.414; *p* < 0.001; Figure 3F).

### Functional Enrichment Analysis

**FIGURE 4:**
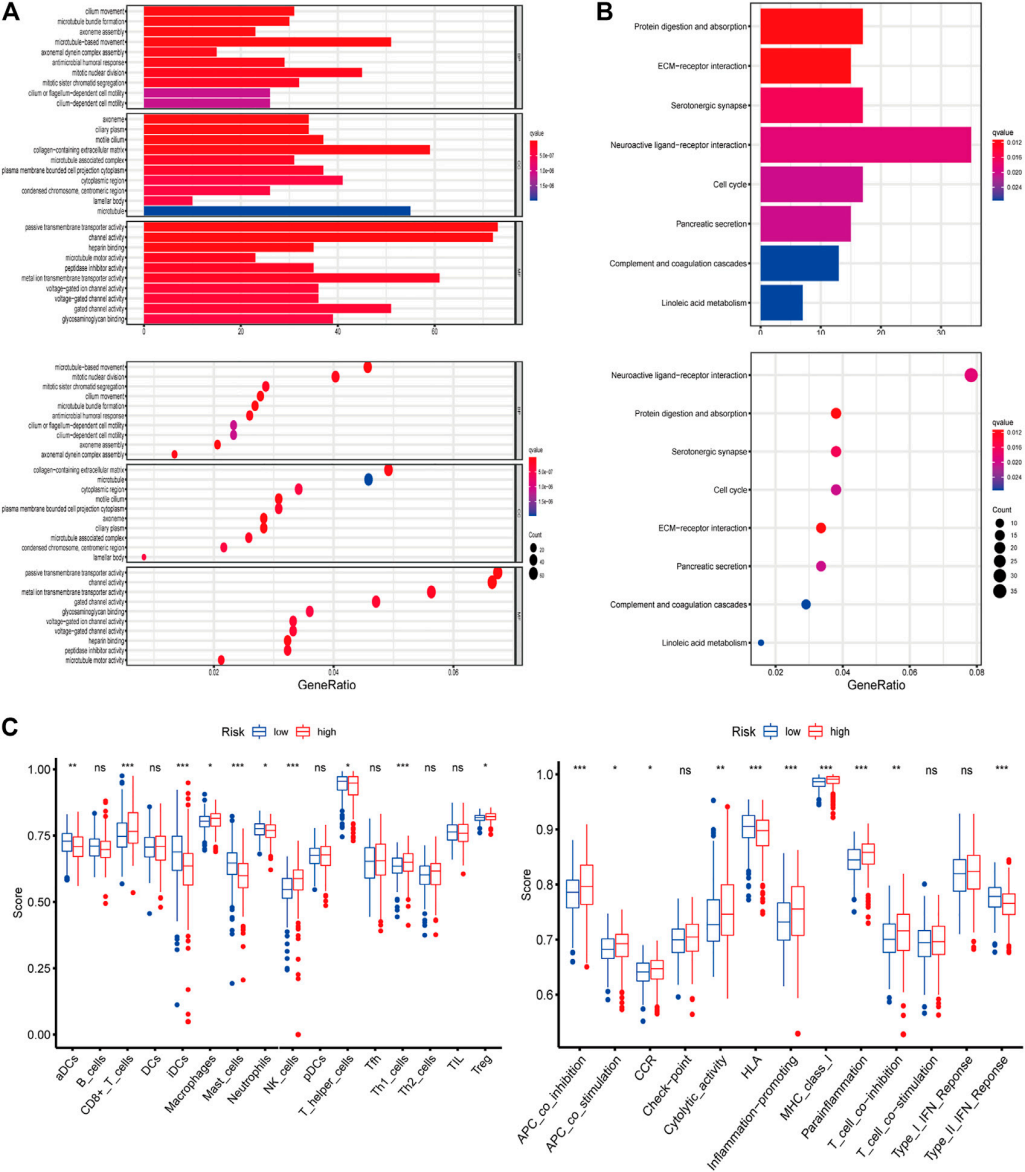
**(A–B)** Representative results of GO and KEGG of the ferroptosis-related genes signature. **(C)** Results of ssGSEA analysis.

The functions and pathways associated with the risk score were elucidated using GO enrichment and KEGG pathway analyses. As expected, DEGs were enriched in several iron-related molecular functions, such as channel activity and metal ion transmembrane transporter activity (*p*-value adjust <0.05; Figures 4A,B). To explore the association between risk score and immune status, we quantified enrichment scores for different immune cell subsets, related functions, or pathways using ssGSEA (Figure 4C). The differences in HLA, MHC class I, type II IFN response, inflammation-promoting macrophages, and Treg cells between the two risk groups were verified by comparisons (adjusted *p* < 0.05). The findings prompted us to investigate whether ferroptosis-related genes were associated with immune cell infiltration.

### The Composition of Tumor-Infiltrating Immune Cells in LUAD

The composition of significant tumor-infiltrating immune cells in the normal and LUAD tissue was assessed using the CIBERSORT algorithm. Macrophages, monocytes, CD4 T cells, plasma cells, mast cells, and neutrophils were highly expressed in tumor and adjacent nontumorous tissues (Supplementary Figures 6A,B), suggesting that they may play essential roles in lung adenocarcinoma. The co-expression analysis was performed using the co-expression correlation among different prognostic immune infiltrating cells (Supplementary Figure 6C). Then, the Wilcoxon rank-sum test indicated that naïve B cells, plasma cells, CD4 T cells, regulatory T cells (T regs), resting NK cells, monocytes, macrophage M0, macrophage M1, macrophage M2, resting mast cells, eosinophils, and neutrophils had significant differences in the immune cell fractions between tumor tissues and adjacent normal tissues in LUAD (Supplementary Figure 6D).

### Clinical Correlation of Tumor-Infiltrating Immune Cells and Nomogram Multiple Cox Risk Regression Analysis

**FIGURE 5:**
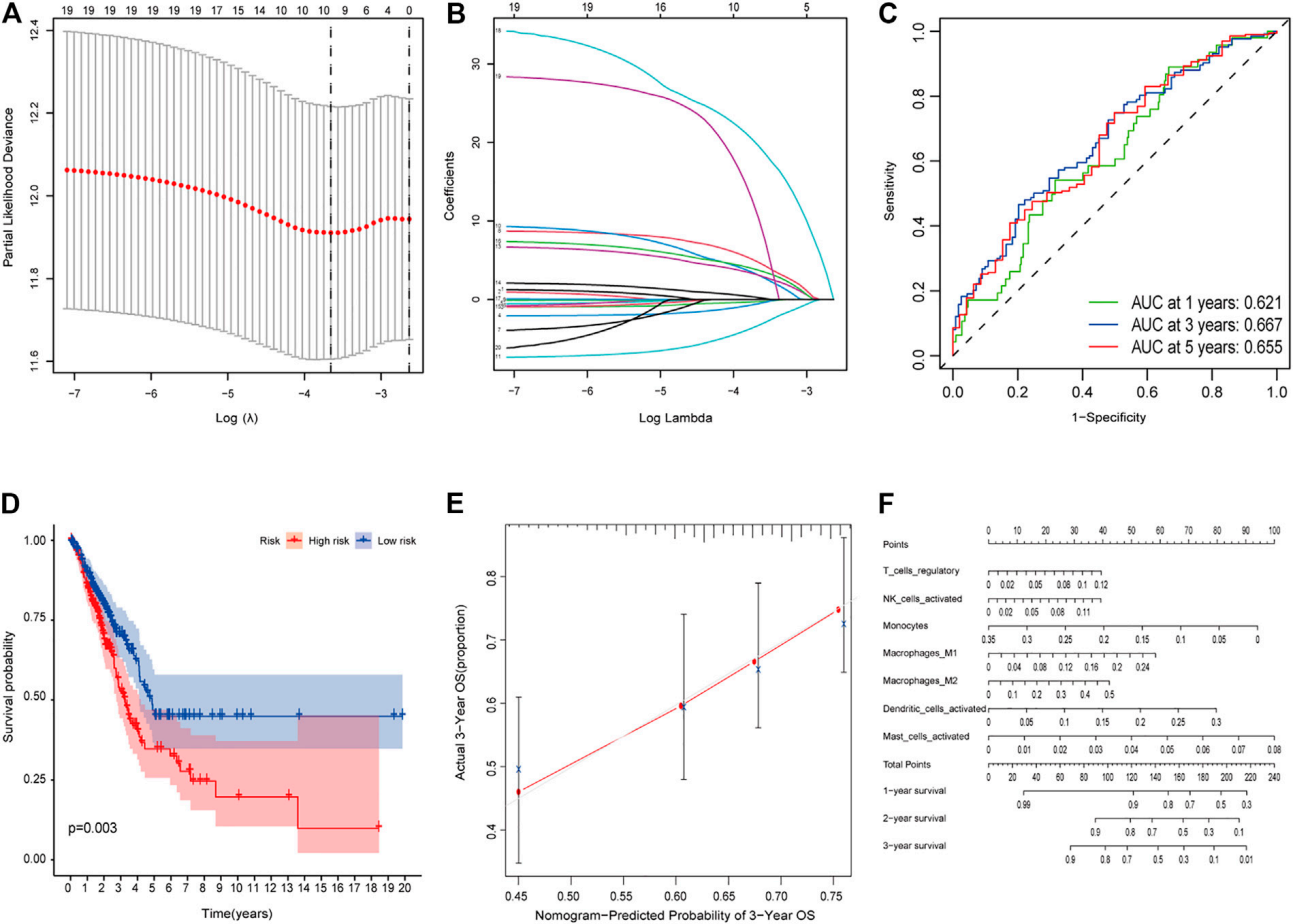
**(A–E)** Model diagnosis process Lasso regression and ROC curves which were constructed by patterns of tumor-infiltrating immune cells. **(F)** Nomogram. ROC, receiver operating characteristic curve. *p < 0.05.

We analyzed the prognosis of 22 types of infiltrating immune cells, focusing on the immune cells that had significant correlation with clinical features (Supplementary Figures 7A–D). The survival analysis results showed that the samples with a low proportion of mast cells were activated, and regulatory T cells (Tregs) had better survival status (log rank test, *p* < 0.05; Supplementary Figures 7E,F). Besides, we used multivariate Cox regression analysis to construct a model that included all significant tumor immune cells, and results showed that the model composed of regulatory T cells, activated NK cells, monocytes, M1 macrophages, M2 macrophages, activated dendritic cells, and activated mast cells (Figures 5A,B). The Kaplan–Meier survival curve suggested that the risk score had a significant prognostic value (*p* = 0.003) (Figure 5D). The nomogram was reliable and accurate according to the ROC curve and calibration curve analysis, with AUC values of 0.621, 0.667, and 0.655 for 1-, 3-, and 5-year survival, respectively (Figure 5C). Then, we utilized a nomogram to show the effects of seven kinds of vital immune cells on 1-year, 2-year, and 3-year overall survival probability based on the multivariable model (Figures 5E,F). In addition, decision curve analysis (DCA) for the immune cell model at 3 years was applied to assess the clinical utility (Supplementary Figure 8).

### Co-Expression of Tumor-Infiltrating Immune Cells and the Ferroptosis-Related Genes

**FIGURE 6:**
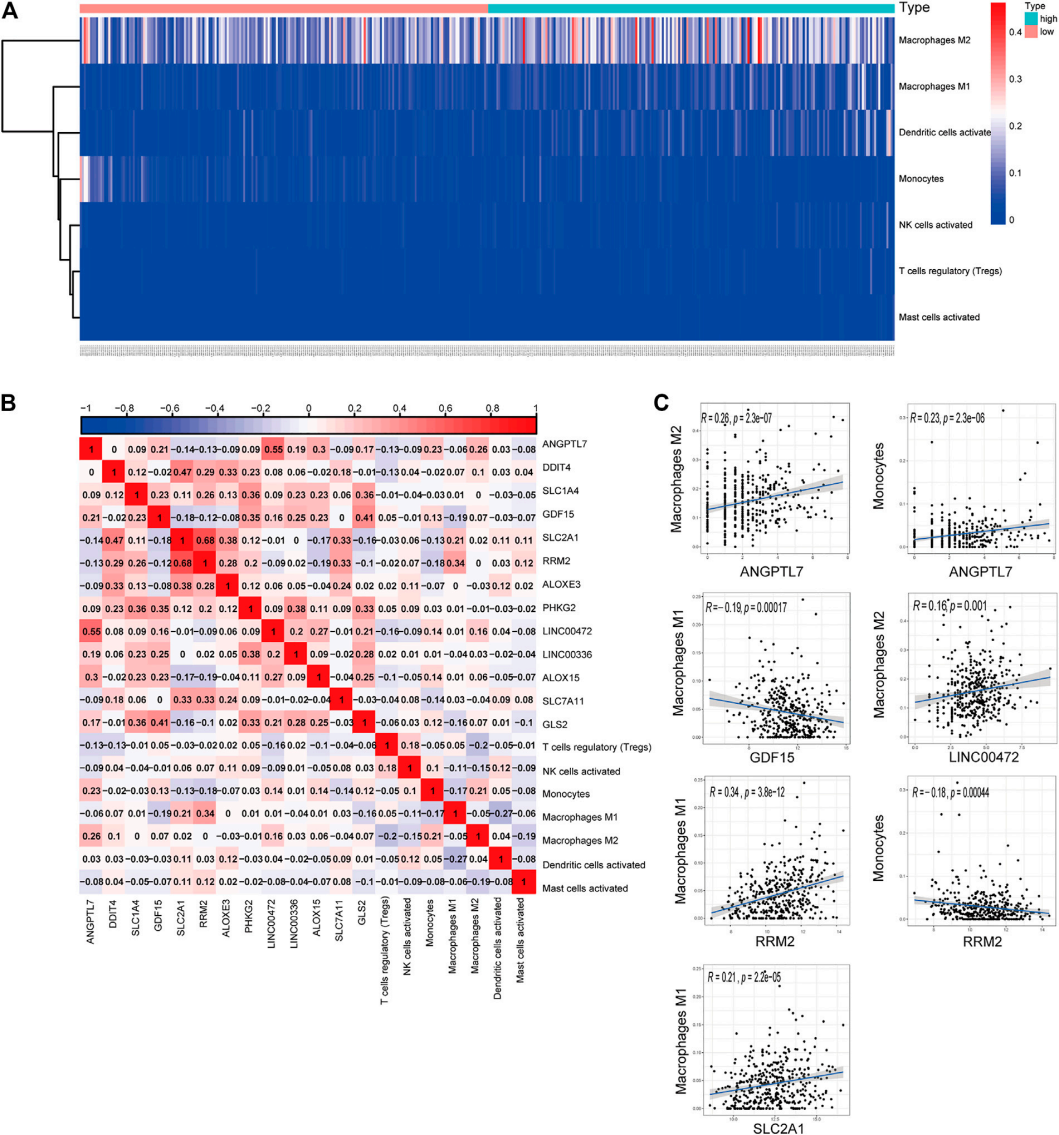
**(A)** The risk heat map of significant infiltrating immune cells. **(B)** The result of the co-expression analysis between tumor-infiltrating immune cells and the ferroptosis-related genes signature. **(C)** ANGPTL7 and M2 macrophages (R = 0.26, *p* < 0.001), ANGPTL7 and monocytes (R = 0.23, *p* < 0.001), GDF15 and M1 macrophages (R = −0.19, *p* < 0.001), LINC00472 and M2 acrophages (R = 0.16, *p* = 0.001), RRM2 and M1 macrophages (R = 0.34, *p* < 0.001), RRM2 and monocytes (R = −0.18, *p* < 0.001), and SLC2A1 and M1 macrophages (R = 0.21, *p* < 0.001), and these reminded us the relationship between the ferroptosis-related genes and tumor-infiltrating immune cells.

We generated a risk heat map (Figure 6A) based on the risk score of infiltrating immune cells. Significant co-expression patterns between prognostic tumor-infiltrating immune cells and key ferroptosis-related genes are shown in Figure 6B. The relationship between the ferroptosis-related genes and tumor-infiltrating immune cells was revealed by ANGPTL7 and M2 macrophages (R = 0.26, *p* < 0.001), ANGPTL7 and monocytes (R = 0.23, *p* < 0.001), GDF15 and M1 macrophages (R = −0.19, *p* < 0.001), LINC00472 and M2 macrophages (R = 0.16, *p* = 0.001), RRM2 and M1 macrophages (R = 0.34, *p* < 0.001), RRM2 and monocytes (R = −0.18, *p* < 0.001), and SLC2A1 and M1 macrophages (R = 0.21, *p* < 0.001; Figure 6C).

## Discussion

Non–small-cell lung cancer is the most prevalent malignant tumor in humans (11.6% of the total cases) and is the primary cause of cancer deaths (18.4% of the total cancer deaths). Previous studies have linked iron dysregulation to lung cancer and poor patient survival (Brooks et al., 2016). Furthermore, the majority of epidemiological data and many experimental studies indicate a close association between iron and lung cancer (Torti et al., 2018; Ward et al., 2019). Consequently, iron synthesis has a significant effect on the tumor microenvironment; thus, tumor cells contain more iron than normal cells (Pfeifhofer-obermair et al., 2018). Previous research has suggested that ferroptosis, a new form of regulated cell death (RCD) that is closely linked to excess iron loading, could play an important role in antitumor immunity and tumor suppression (Jiang et al., 2015; Friedmann et al., 2019). In addition, several studies have proposed that ferroptosis and immune infiltration are important in tumorigenesis and growth, but the specific mechanism remains unknown.

In this study, 13 ferroptosis-related genes were used to create a new prognostic model. Some of these genes had been shown to play different roles in immune infiltration in previous studies. For example, it was reported that SLC2A1 mediates glucose uptake and affects macrophage activation (Freemerman et al., 2019), which was confirmed in our finding that SLC2A1 is correlated with M1 macrophage. Inflammation associated with monocyte/macrophage cells was promoted by ANGPTL7 through the mitogen-activated protein kinase (MAPK) signaling pathway (Qian et al., 2016). Dual oxidase (DUOX) 1, a transmembrane enzyme, plays a critical role in limiting macrophage antitumor activity (Meziani et al., 2020).

The other genes were involved in iron metabolism and ferroptosis, such as the genes LINC00472, DPP4, DDIT4, and GLS2, and may play important roles in p53-mediated ferroptosis (Basu et al., 2016; Xie et al., 2017). Genes GDF15, SLC7A5, and SLC7A11 protect cells from Golgi stress and prevent cell death during ferroptosis (Alborzinia et al., 2018; Chen et al., 2020; Hong et al., 2021). Gene SLC2A4 appears to be associated with iron metabolism. Inhibition of aurora kinase A (AURKA) can suppress ferroptosis in upper gastrointestinal cancers (Gomaa et al., 2019), and RRM2 protects against ferroptosis in liver cancer (Yang et al., 2020). Silencing TXNRD1 was proven to enhance cytotoxicity of triple-negative breast cancer cells (Chepikova et al., 2020). Upregulated arachidonate lipoxygenase 3 (ALOXE3) can promote ferroptosis in colorectal cancer (Xia et al., 2020). Iron metabolism is regulated by phosphorylase kinase G2 (PHKG2) and cysteine dioxygenase 1 (CDO1), which leads to ferroptosis (Yang et al., 2016; Hao et al., 2017). The LINC00336 gene suppresses ferroptosis in lung cancer by functioning as a competing endogenous RNA (Wang et al., 2020). Arachidonate lipoxygenase 15 (ALOX15) is linked to lipid-ROS synthesis in gastric cancer (Zhang et al., 2020). Finally, the glutamate–cysteine ligase catalytic subunit (GCLC) can protect against ferroptosis in lung cancer (Kang et al., 2021).

In this study, we investigated 213 ferroptosis-related genes in LUAD tumor tissues and screened 31 genes correlations with OS (Supplementary Figure 1). We also constructed a novel prognostic model that integrated 13 ferroptosis-related genes, as well as 22 types of tumor-infiltrating immune cells. Strikingly, we found that ferroptosis had a significant impact on macrophage activation and CD8 T-cell function in both (ssGSEA and Cibersort) analyses of infiltrating immune cells. Moreover, the interactions between ANGPTL7 and M2 macrophages, ANGPTL7 and monocytes, GDF15 and M1 macrophages, LINC00472 and M2 macrophages, RRM2 and M1 macrophages, RRM2 and monocytes, and SLC2A1 and M1 macrophages were statistically significant in the co-expression analysis between tumor-infiltrating immune cells and ferroptosis-related genes. We verified their connection again in the TIMER database (Li et al., 2020) (Supplementary Figure 5). Thus, we deduced that the abovementioned seven pairs, and their underlying mechanisms, play important roles in the prediction and treatment of LUAD.

Compared with previous prognostic models (Shi et al., 2020; Gao et al., 2021), the current prognostic model could provide significantly better performance. And our prognostic models could provide individualized mortality risk prediction, which were of great significance for clinical application by clinicians. Our study inevitably has limitations. The data in our research are entirely from TCGA database and GEO datasets, implying that the clinical utility of our model needs more prospective real-world data to be verified. Furthermore, there was no experimental evidence that associated risk sore with immune activity.

To summarize, we defined a prognostic model of 13 ferroptosis-related genes, and identified seven pairs of co-expressed ferroptosis-related genes and tumor-infiltrating immune cells. Our study provides insight into the underlying mechanism between ferroptosis and tumor-infiltrating immune cells in LUAD.

## Data Availability

The datasets presented in this study can be found in online repositories. The names of the repository/repositories and accession number(s) can be found in the article/Supplementary Material.
