# Supplementary figures and images for: Ferroptosis-Related Gene Signature and Patterns of Immune Infiltration Predict the Overall Survival in Patients With Lung Adenocarcinoma
Source: Front Mol Biosci. 2021 Jul 30;8:692530. doi: 10.3389/fmolb.2021.692530 (PMC8360867; doi:10.3389/fmolb.2021.692530)

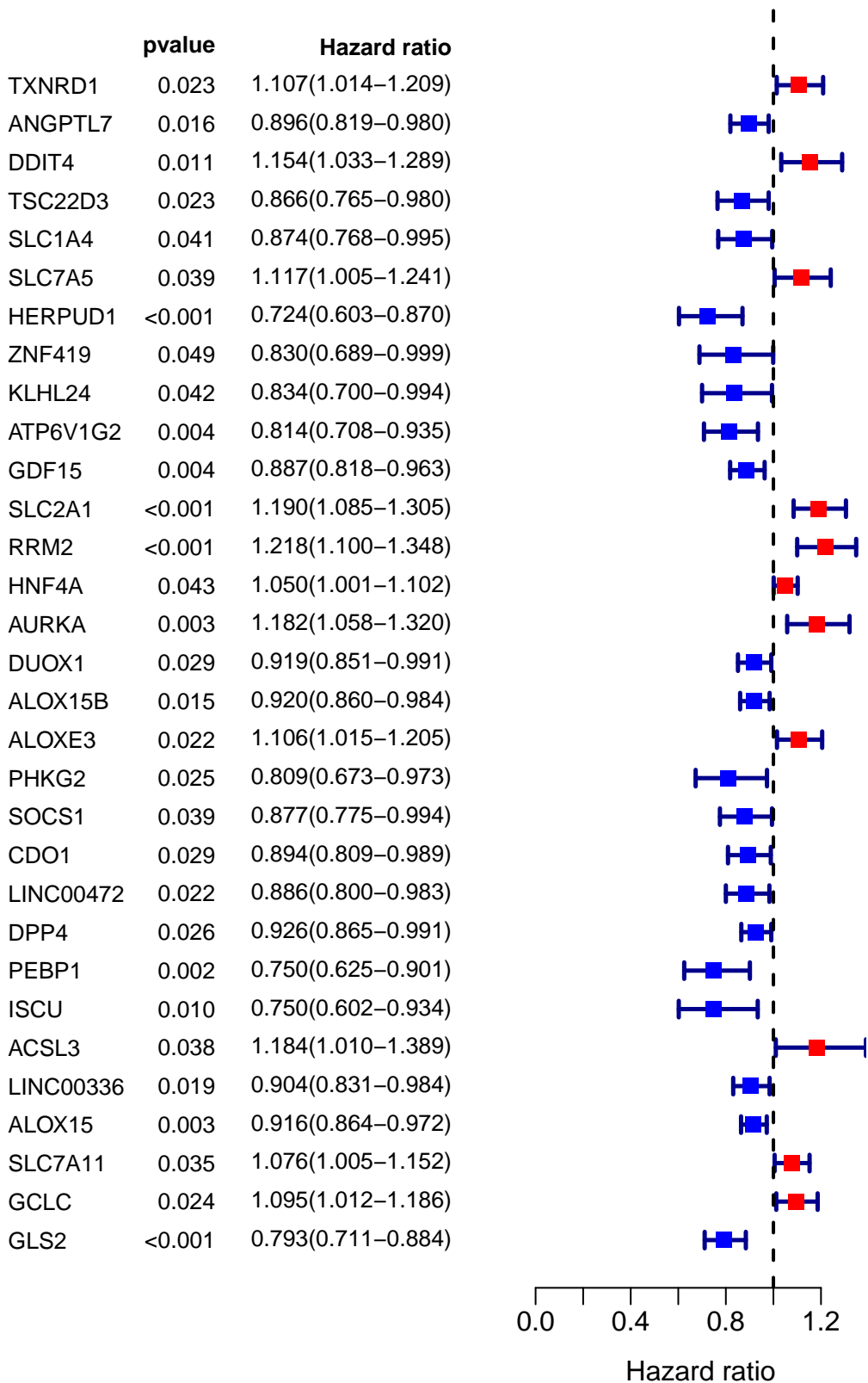

Supplement: Supplementary file 1 [file DataSheet2.PDF]

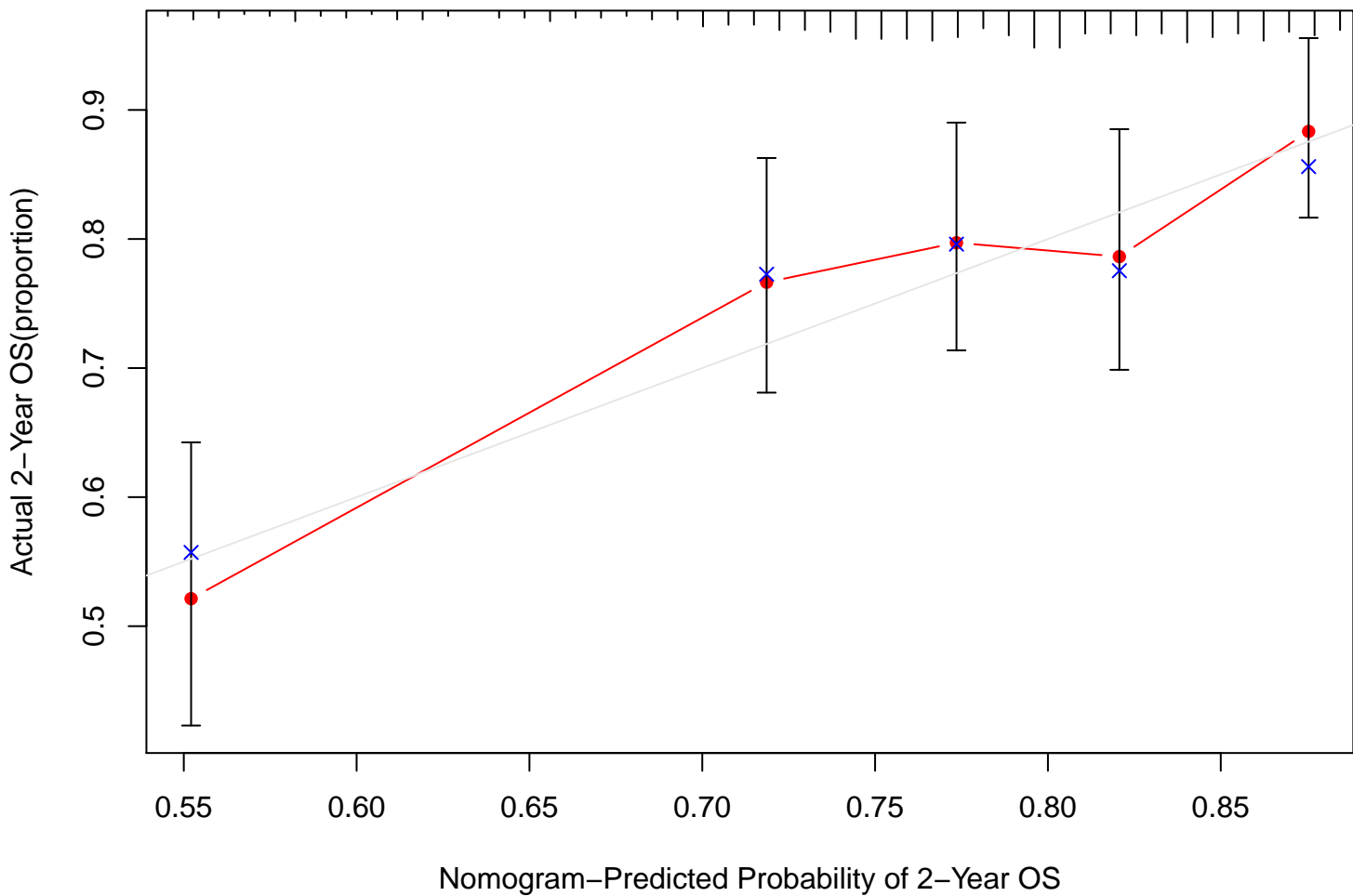

Supplement: Supplementary file 2 [file DataSheet4.PDF]

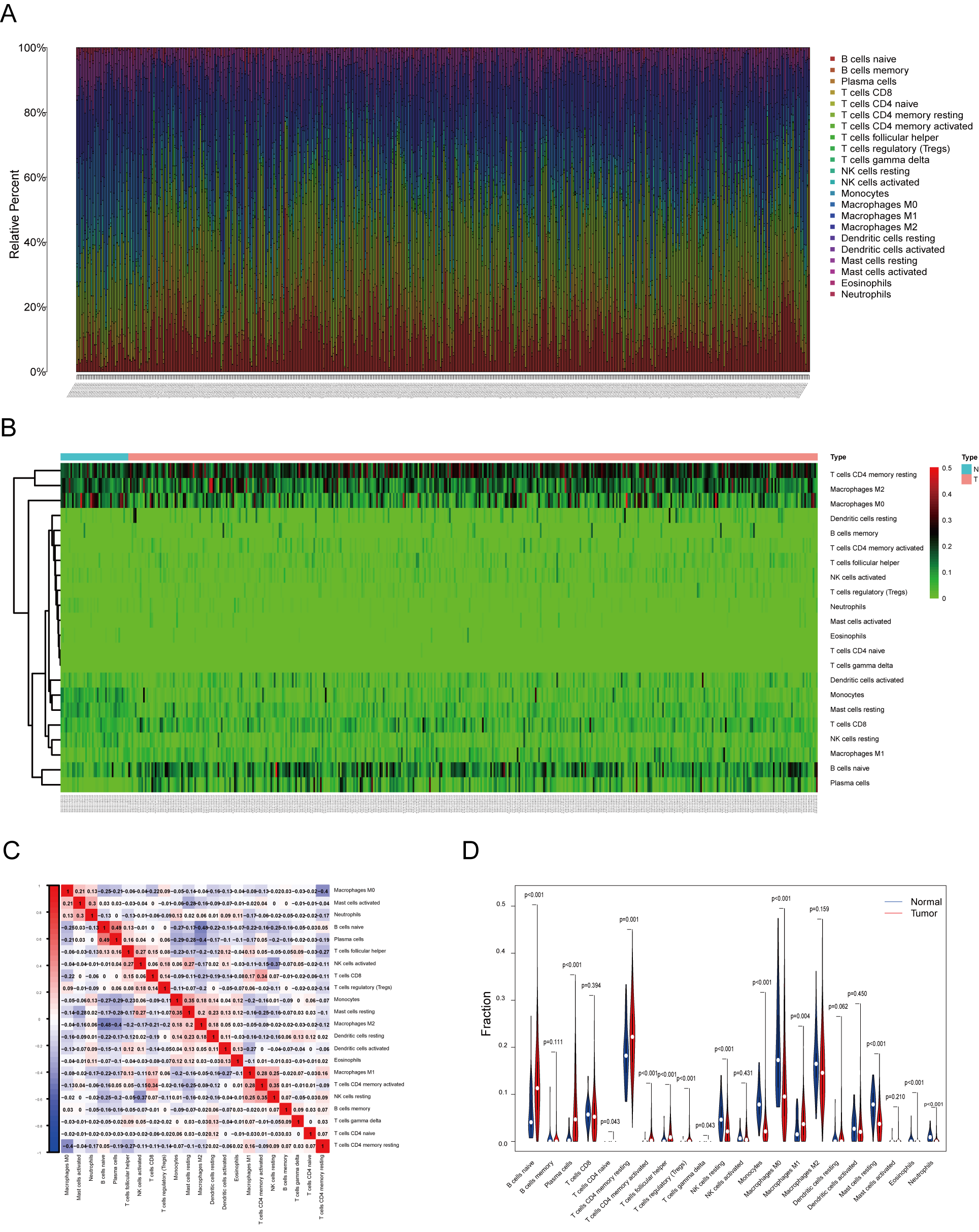

Supplement: Supplementary file 5 [file Image3.TIF]

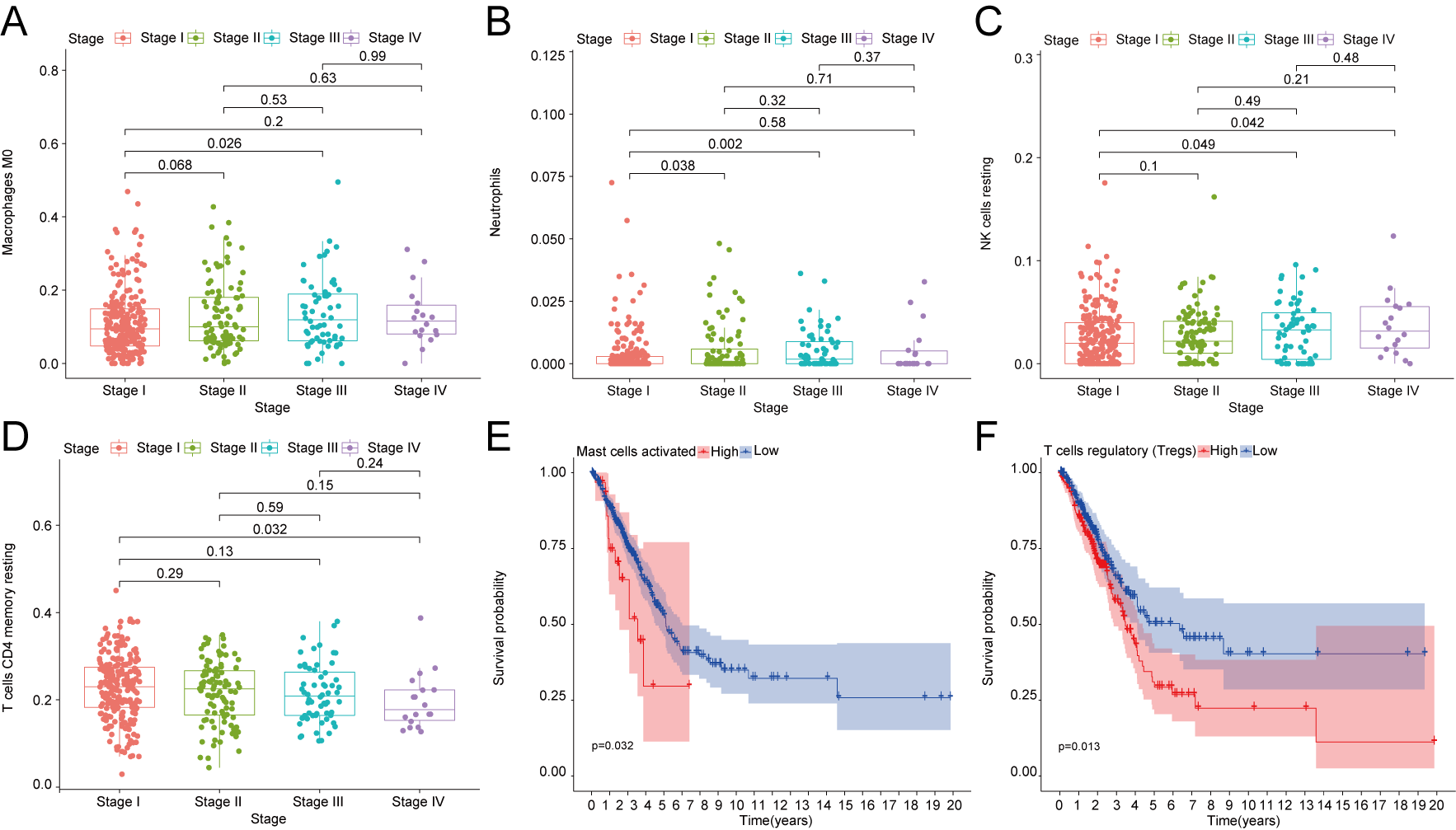

Supplement: Supplementary file 6 [file Image4.TIF]

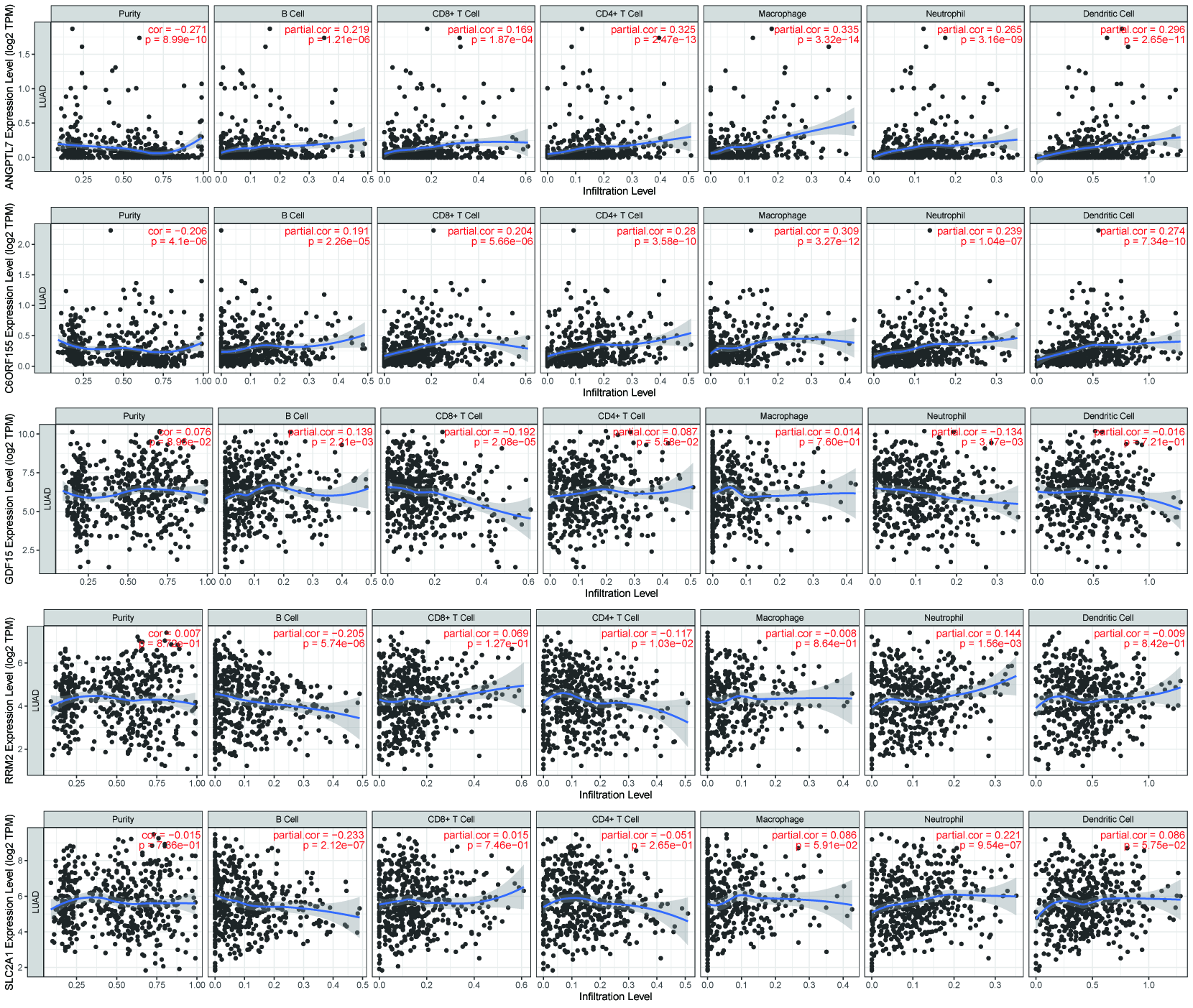

Supplement: Supplementary file 7 [file Image2.TIF]

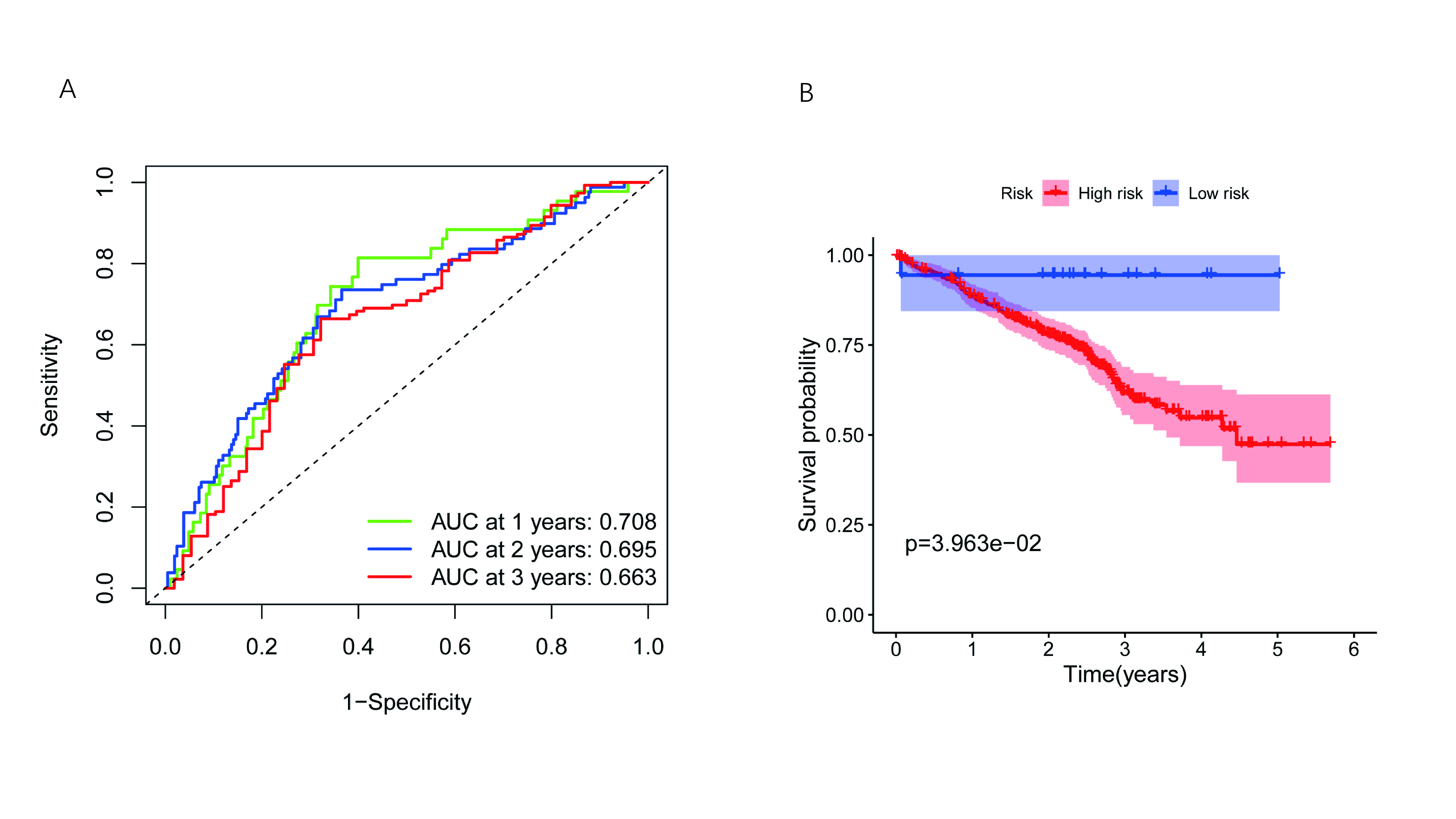

Supplement: Supplementary file 8 [file Image1.TIF]

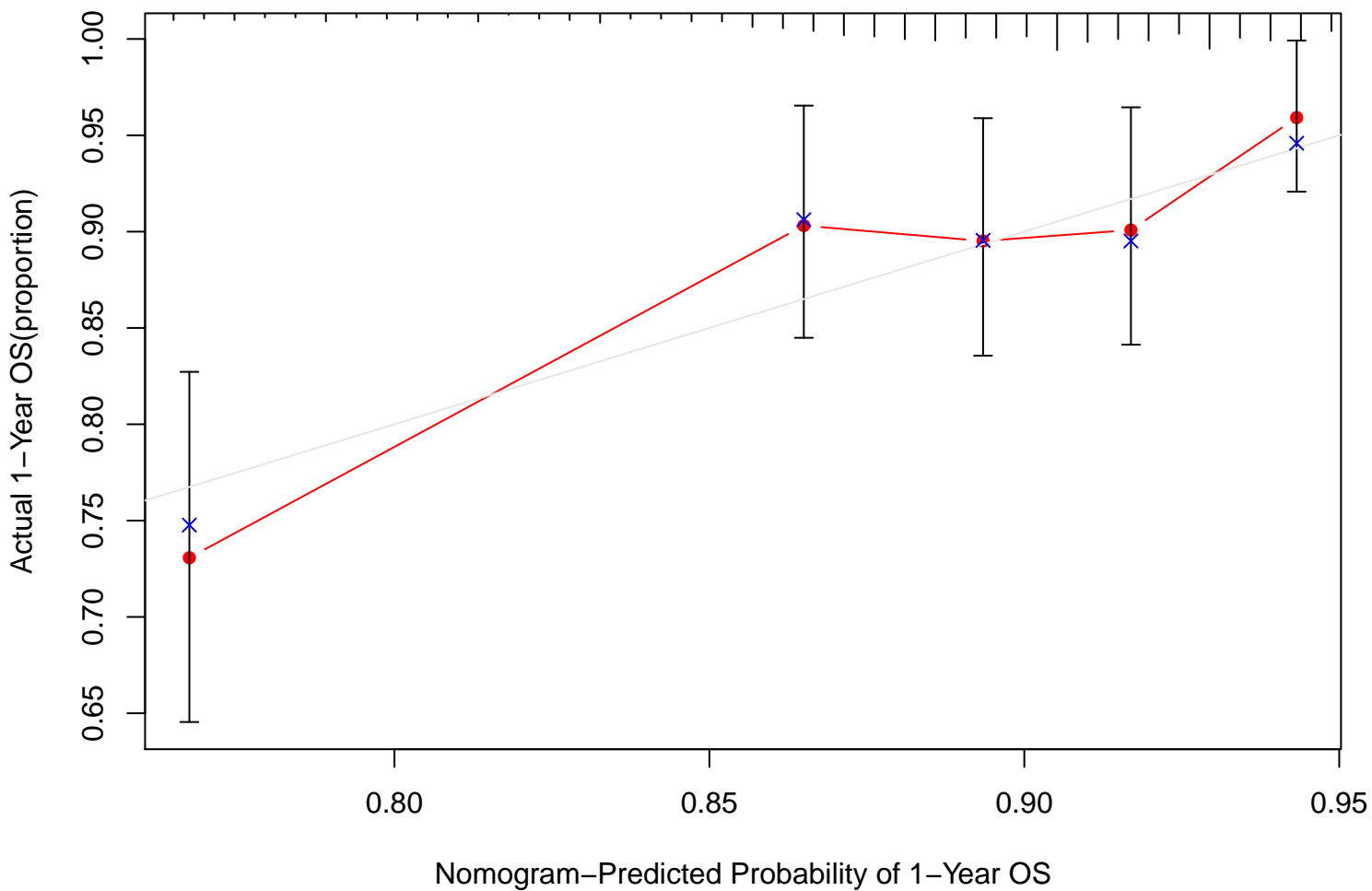

Supplement: Supplementary file 9 [file DataSheet3.PDF]

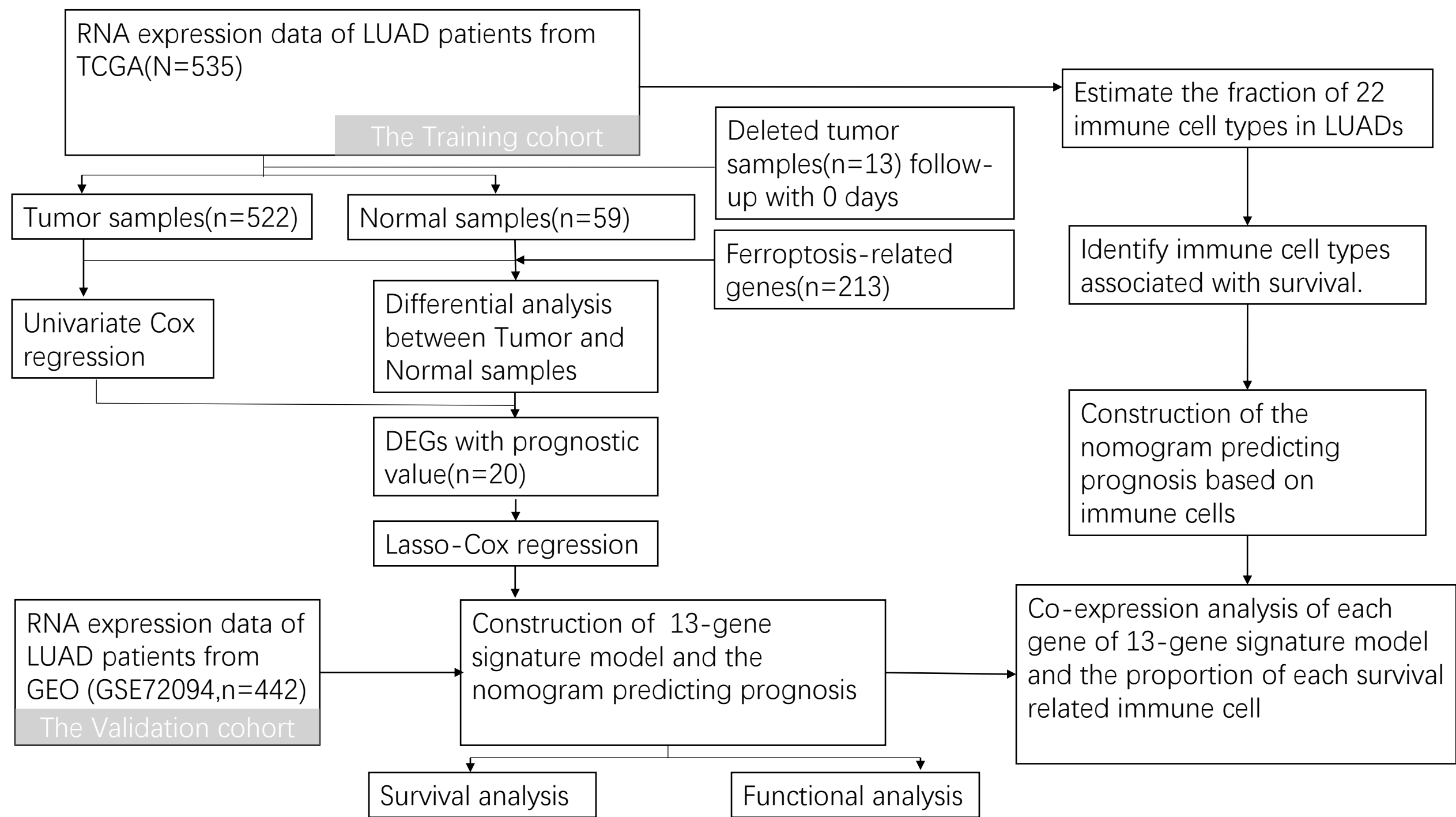

Supplement: Supplementary file 10 [file DataSheet1.PDF]

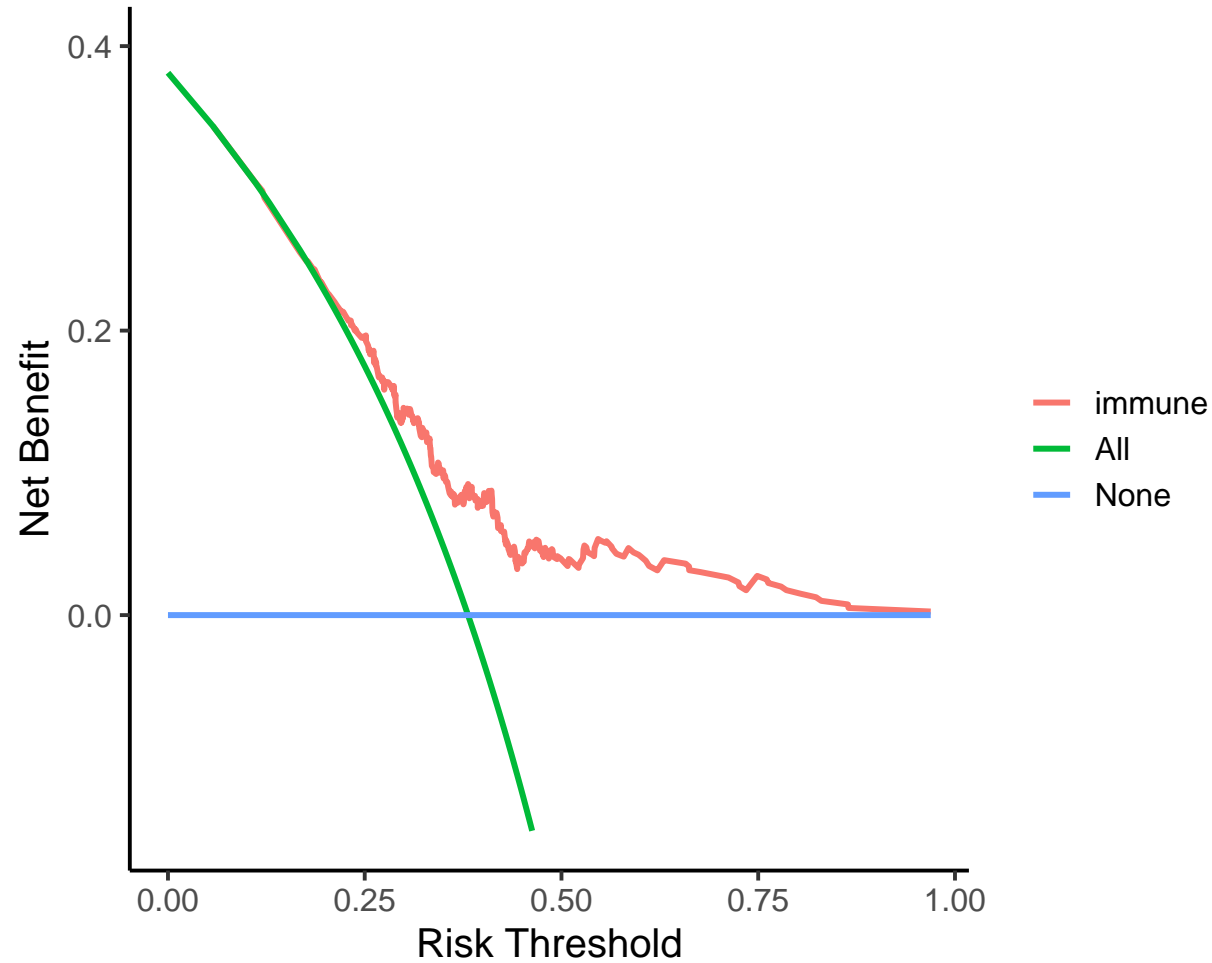

Supplement: Supplementary file 11 [file DataSheet5.PDF]
