# Supplementary material for: Ferroptosis-Related Gene Signature and Patterns of Immune Infiltration Predict the Overall Survival in Patients With Lung Adenocarcinoma
Source: Front Mol Biosci. 2021 Jul 30;8:692530. doi: 10.3389/fmolb.2021.692530 (PMC8360867; doi:10.3389/fmolb.2021.692530)
Supplement: Supplementary file 12 [file Table3.DOCX]

**Figure Legends**

**Figure S1.** 31 prognostic ferroptosis-related genes of LUAD.

**Figure S2.** A. The time-dependent ROC curves and the area under the curve (AUC); B. The Kaplan-Meier curve. *P < 0.05.

**Figure S3.** The calibration curve of 1-year

**Figure S4.** The calibration curve of 2-year

**Figure S5.** Correlation between tumor-infiltrating immune cells and the ferroptosis-related genes from the external database TIMER.

**Figure S6.** (A-B) The composition of immune cells assessed by CIBERSORT algorithm in lung adenocarcinoma. (C) The result of the co-expression analysis between significant immune cells. (D) The identification of prominent tumor-infiltrating immune cells related to lung adenocarcinoma.

**Figure S7.** (A-D) Four significant immune cells correlated with stage. (E-F) The result of the Kaplan-Meier curves of Mast cells activated and T cells regulatory (Treg).

**Figure S8.** Decision curve analysis (DCA) of Ferroptosis related prognostic model.

**Table Legends**

**Table S1.** Ferroptosis related genes retrieved from previous literature and databased.

**Table S2.** DEGs with log2FC and adj-p-value
